# Metformin treatment response is dependent on glucose growth conditions and metabolic phenotype in colorectal cancer cells

**DOI:** 10.1038/s41598-021-89861-6

**Published:** 2021-05-18

**Authors:** Abdelnour H. Alhourani, Tia R. Tidwell, Ansooya A. Bokil, Gro V. Røsland, Karl Johan Tronstad, Kjetil Søreide, Hanne R. Hagland

**Affiliations:** 1 Department of Chemistry, Bioscience and Environmental Engineering, University of Stavanger, Stavanger, Norway; 2 Department of Biomedicine, University of Bergen, Bergen, Norway; 3 Department of Oncology and Medical Physics, Haukeland University Hospital, Bergen, Norway; 4 Department of Gastrointestinal Surgery, Stavanger University Hospital, Stavanger, Norway

**Keywords:** Cancer, Cancer metabolism

## Abstract

Cancer cells exhibit altered metabolism, a phenomenon described a century ago by Otto Warburg. However, metabolic drug targeting is considered an underutilized and poorly understood area of cancer therapy. Metformin, a metabolic drug commonly used to treat type 2 diabetes, has been associated with lower cancer incidence, although studies are inconclusive concerning effectiveness of the drug in treatment or cancer prevention. The aim of this study was to determine how glucose concentration influences cancer cells’ response to metformin, highlighting why metformin studies are inconsistent. We used two colorectal cancer cell lines with different growth rates and clinically achievable metformin concentrations. We found that fast growing SW948 are more glycolytic in terms of metabolism, while the slower growing SW1116 are reliant on mitochondrial respiration. Both cell lines show inhibitory growth after metformin treatment under physiological glucose conditions, but not in high glucose conditions. Furthermore, SW1116 converges with SW948 at a more glycolytic phenotype after metformin treatment. This metabolic shift is supported by changed GLUT1 expression. Thus, cells having different metabolic phenotypes, show a clear differential response to metformin treatment based on glucose concentration. This demonstrates the importance of growth conditions for experiments or clinical studies involving metabolic drugs such as metformin.

## Introduction

Nearly a century ago, Otto Warburg described a commonality among many cancers that is still under intense study^1^. What Warburg described was the cancer cells’ ability to consume glucose, even in the presence of oxygen, later termed the Warburg effect. Cancer cells that have perfected this ability are avid glucose consumers supporting a high proliferation rate, with many cell signalling pathways primed to maintain this rapid growth^2^. Thus, many studies show that calorie restriction and nutrient deprivation may be both cancer preventative and may enhance treatment response^3^. However, lowering blood glucose within fasting range (< 5 mmol/L) seems to not be enough to prevent cancer growth, as cancer cells express high affinity glucose receptors^4–6^, which even at fasting glucose levels, as low as 1 mmol/L, are able to import glucose^7^. However, lowering glucose levels may compromise the metabolic flexibility of these cells under stress^8^. Therefore targeting cancer metabolism is an interesting avenue to pursue, and has prompted many recent studies testing the efficacy of metabolic drugs for cancer treatment^9^. One drug that has spurred great interest is metformin, normally used to treat type 2 diabetes (T2D). Metformin use in diabetes is associated with lower incidence in many cancer groups worldwide compared to diabetic patients not using metformin^10–13^. It has since been studied in pre-clinical settings using in vitro cancer cell models^14–16^, animal models^17–21^, and consequently over 300 clinical trials are found when searching “metformin AND cancer” in clinicaltrial.gov. The high number of initiated clinical trials reflect both the low cost of the drug and the extensive use with minimal side effects reported since it was approved as a drug nearly 60 years ago^22,23^. However, many of these studies have so far been inconclusive, and have not shown major treatment effects nor improved survival^23–26^. The low success rate may be due to many reasons, but lack of patient stratification, metformin dosage, and mode of delivery seem to be areas to address. A regular metformin treatment in diabetic patients starts at 500–850 mg administered orally every 12 h, increasing to, but no more than 2550 mg/day, achieving a steady state concentration of 1.4 mg/L (10.8 µM)^27^. As an orally ingested drug, its highest concentration is found in the gastrointestinal (GI) tract, and GI derived tumours would most likely be affected^28^, which further indicates that achieved drug concentration play a major role in tumour responses. This is recapitulated in its various mode of action ranging from influence on microbiota^29^, immune modulation^30^, and direct intracellular effect^31^.

Metformin became an interest in the cancer field due to studies reporting that diabetic colorectal cancer (CRC) patients taking metformin had a better overall survival compared to those treated with other glucose lowering drugs^14,26,32,33^. This effect has largely been tied to the lowering of insulin growth factor and activation of AMPK^22,34–36^. Hence, the potential anticancer effect of metformin is most likely not due to expected lowering of blood glucose, but rather due to intracellular effects of metformin in the tumour cells^37^. Once inside the cell, the exact target of metformin has been difficult to pinpoint, although a change in mitochondrial function is one major effect seen^34–36^, attributed to its inhibition of the electron transport chain (ETC)^37^. Metformin has also been shown to reduce GLUT1 expression alongside HIF-1α^38^. GLUT1 is a rate limiting transporter for glucose metabolism and involved in maintaining higher levels of glycolysis intermediates^39,40^. Clinically, its increased expression has been correlated with tumour aggressiveness and is correlated with increased proliferation activity and poor survival^5^. However, this increase could be either due to an oncogenic transformation in cells or indirectly due to high glucose consumption of cancer cells and resulting low intracellular glucose levels^40^. On the other hand, reduced glycolysis due to lower levels of GLUT1 has been associated with less malignancy^40^. Monitoring GLUT1 levels under the previously mentioned conditions could be an indicator of the adaptive mechanisms different metabolic phenotypes undergo in their response to glucose levels and metformin treatment.

In the last decades, there have been a few major breakthroughs in cancer drug discovery^41^, however a fundamental issue for drug development is the discrepancy in drugs’ effects once they reach efficacy testing in humans, despite promising results in earlier pre-clinical model systems^42^. One major challenge relates to the unphysiological metabolic conditions normally applied in cell cultures, often using supraphysiological levels of glucose in the growth medium. Moreover, most studies investigating metformin have applied higher concentrations than what is achievable in vivo^14,34,37,43^. Metformin is administered orally, typically achieving concentrations at up to 300 times higher in the GI tract than that found in plasma^44^. The aim of this study was therefore to test how colorectal cancer cells responded to metformin at a concentration typically found in the GI tract and grown in physiological glucose media. We show that using physiologically relevant glucose levels in cell growth media could provide a response to metformin that is more representative to in vivo conditions and that GLUT1 may be used as a metabolic biomarker for studying these responses.

## Results

### Glucose concentration in culture media affect cellular proliferation rates

**Figure 1:**
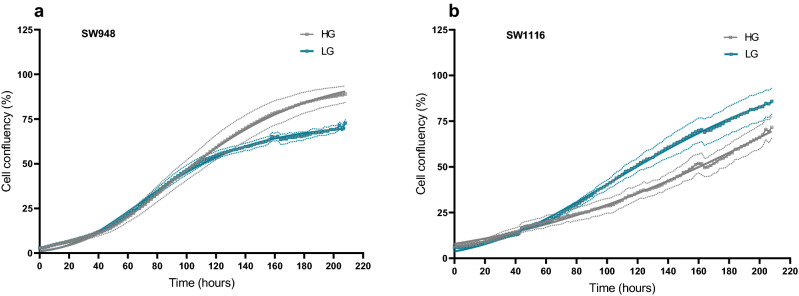
Cell growth recording of SW948 and SW1116 in HG and LG over 9 days continuous monitoring using Incucyte ZOOM. Cell confluency was analyzed in the Incucyte ZOOM software and plotted here as confluency (%) over time (hours). (**a**) SW948, initial seeding density of 10,000 cells/well. (**b**) SW1116, initial seeding density of 20,000 cells/well. Complete growth medium was exchanged at day 2 and 7 of the culture period. Grey solid line depicts growth in high glucose (HG) conditions, whereas blue solid line represents growth in low glucose conditions (LG). The dotted lines represent standard deviation from the average of N = 8 for each condition.

A glucose concentration of 25 mmol/L is commonly used for in vitro cell culture, and this is from here-on referred to as high glucose (HG). The physiological glucose concentration in blood at fasting state is around 5 mmol/L, here referred to as low glucose (LG). We found that glucose concentration in the growth media directly affects the proliferation rates of two cell lines SW948 (Fig. 1a) and SW1116 (Fig. 1b) with differential metabolic phenotypes (Fig. 3). By culturing them in HG and LG over 9 days with two full media changes (day 2 and 7), we found that the doubling time of SW948 is 25.1 ± 1.2 h and 25.0 ± 0.4 h in HG and LG, respectively. Whilst for SW1116 the doubling time is 74.0 ± 10.1 h in HG and 41.8 ± 2.4 h in LG (p < 0.0001). The glucose effect is not immediate in either cell line, where in SW948 the effect on proliferation becomes apparent only when the stationary growth phase begins, thus showing lower cell confluency in LG. In contrast, glucose has a significant effect on the exponential growth of SW1116, with low glucose conditions resulting in increased proliferation.

### Metformin predominantly suppressed the proliferation of SW948 and SW1116 cells under physiological glucose conditions in comparison to high glucose culture conditions

**Figure 2:**
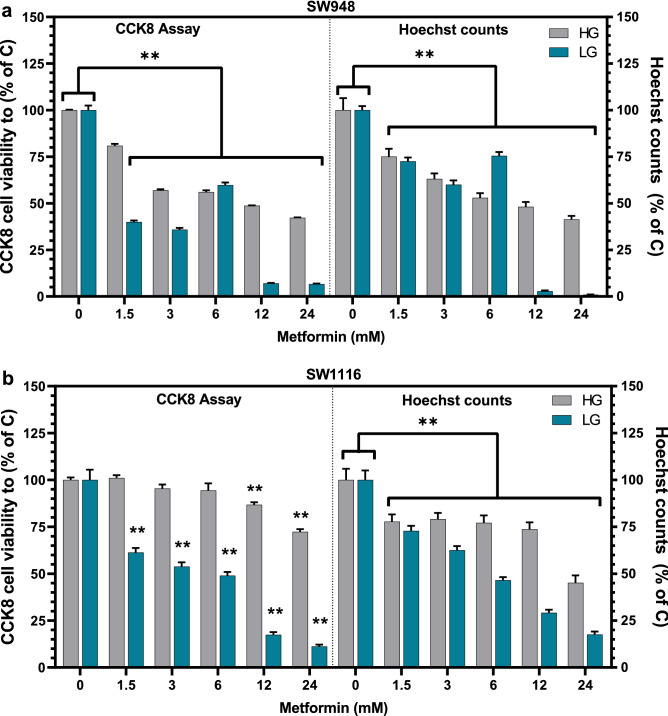
Viability assays of SW948 and SW1116 after metformin treatment for 48 h. The effect on the viability of (**a**) SW948 cells and (**b**) SW1116 in HG (25 mmol/L) and LG (5 mmol/L) is presented. Viability was calculated using CCK-8 (left) and cell counts were scored using fluorescent microscopy on Hoechst stained cells (right). CCK-8 absorbance values and the calculated cell numbers for all metformin treatments (1.5–24 mM) were compared to high glucose control (25 mmol/L, 0 mM Metformin) to show relative viability and cell numbers. Error bars denotes s.e.m, and statistical analysis was calculated using two-way ANOVA (** is p < 0.05) in GraphPad Prism (N = 3). *C* Control, *HG* high glucose, *LG* low glucose.

To see if the concentration of glucose in the growth media affects the cellular response to metformin treatment, we tested a range of metformin concentrations up to the clinically achievable range and higher only achievable in vitro. Since many proliferation assays measure metabolic activity (Alamar blue, WST-1), which may be affected by metformin treatment directly, the two assays we used were compared to nuclei count for verification of results. We found that after 48 h of metformin treatment, the SW948 cells (Fig. 2a) exhibit a concentration-dependent reduction in viability in HG up to 42% in 24 mM metformin treatment compared to HG control, which is consistent with corresponding cell counts. However, in LG, the CCK-8 measured viability of SW948 is reduced to 40% in comparison to LG control, even at the lowest metformin concentration (1.5 mM). At metformin treatments of 6 mM and lower there are discrepancies between the two viability measurements, whereas both cellular viability and cell numbers show more than 90% reduction in viability to control during metformin treatment of 12 mM.

There is no observed reduction in the viability of SW1116 cells (Fig. 2b) in HG using metformin concentrations up to 6 mM, while 12 and 24 mM concentrations result in viabilities of 86% and 72%, respectively. Cell numbers are lower than control across all treatments but follow the same trends as the CCK-8 assay results. In LG, the metformin effect is again found to be concentration-dependent with a reduction in viability from 61% (1.5 mM) to 11% (24 mM). The reduction in cell numbers is also consistent in a stepwise fashion with the increasing metformin concentrations and corresponds to CCK-8 viability results.

### Metabolic phenotype plays a role in response to metformin

**Figure 3:**
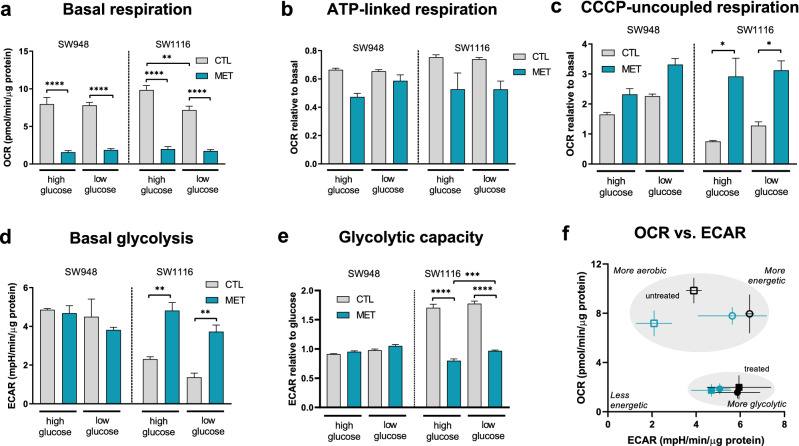
OCR and ECAR were measured in the Seahorse XF96 instrument, with injections of sequential compounds and concentrations according to the assays detailed in the methods section. Mitochondrial stress test assay: (**a**) Basal respiration, before any injections. (**b**) ATP-linked respiration, after oligomycin injection, shown relative to basal OCR level. (**c**) CCCP-uncoupled respiration, shown relative to basal OCR level. Glycolysis stress test assay: (**d**) Basal glycolysis, after glucose injection. (**e**) Glycolytic capacity, after oligomycin injection, shown relative to glycolysis level after glucose injection. Error bars denote s.e.m. Statistical analysis was performed using one-way ANOVA in GraphPad Prism (*p < 0.05; **p < 0.01; ***p < 0.001; ****p < 0.0001) (N = 2–8) (**f**) OCR vs ECAR from the mitochondrial stress test assay. Squares: SW1116; Circles: SW948. Metabolic phenotypes of untreated (open symbols square, circle: control) and treated (closed symbols circle, square: 3 mM metformin) samples. Black: high glucose (25 mmol/L); Teal: low glucose (5 mmol/L). Error bars denote s.d. *OCR* Oxygen concentration rate, *ECAR* extracellular acidification rate, *CTL* control 0 mM metformin, *MET* metformin 3 mM pre-treatment for 48h.

Metformin is thought to affect mitochondrial function, although its specific target in the mitochondria has been a source of debate. To investigate effects of metformin on mitochondrial respiration and glycolysis in the two cell lines, we measured the cellular oxygen consumption rate (OCR) and lactate production assessed via extracellular acidification rate (ECAR). Specific protocols involving sequential addition of modulators were used to test key functions of oxidative phosphorylation in the metabolic flux analyzer Seahorse XFe96 (see “Methods”). In SW948, there is no significant change in oxygen consumption rate (OCR) between high (7.953 ± 0.905 pmol O_2_/min/µg protein) or low glucose (7.791 ± 0.407 pmol O_2_/min/µg protein) medium (Fig. 3a). However, 48 h metformin treatment causes a significant drop in OCR levels in both glucose conditions (HG: 1.571 ± 0.216 pmol O_2_/min/µg protein, p < 0.0001; LG: 1.862 ± 0.182 pmol O_2_/min/µg protein, p < 0.0001) (Fig. 3a). In SW1116 cells there is a significant drop in OCR when cells are grown in low glucose compared to high glucose conditions (9.839 ± 0.598 and 7.175 ± 0.522 pmol O_2_/min/µg protein, respectively, p = 0.0018). Like SW948, the metformin treatment in SW1116 also causes a drop in OCR in these cells under both glucose conditions (HG: 1.979 ± 0.342 pmol O_2_/min/µg protein, p < 0.0001; LG: 1.737 ± 0.179 pmol O_2_/min/µg protein, p < 0.0001) (Fig. 3a). Both cell lines exhibit similar normalized basal respiratory levels in control conditions and after 48 h with metformin treatment (Fig. 3a), when compared to one another. The ATP-linked respiration (Fig. 3b), revealed by oligomycin inhibition of ATP-synthase, was not significantly affected by high and low glucose concentrations in either cell line. While after metformin treatment, this is lower albeit not significant compared to the control. CCCP was used to uncouple the mitochondria and measure respiration under mild stress conditions. This CCCP-uncoupled respiration increases in both cell lines when grown in low glucose media and is further increased after metformin treatment (Fig. 3c). In SW1116, the increase in CCCP-uncoupled respiration (HG: p = 0.0191; LG: p = 0.0314) is significant compared to control. To see if HG or LG media and metformin influenced the cells’ ability to use glycolysis, we performed a glycolysis stress test. We found that in SW948 there is no significant change in the cells’ ability to use glycolysis under any of the conditions nor after metformin treatment (Fig. 3d), while metformin treated SW1116 cells show significantly increased glycolysis compared to control (HG: p = 0.0027; LG: p = 0.0083) (Fig. 3d). To see if either cell line had spare glycolytic capacity, we added oligomycin to block ATP synthesis in the mitochondria. We found that SW948 did not have increased glycolytic capacity, whereas SW1116 cells increased glycolytic capacity by over 170% in both high and low glucose control conditions. However, this glycolytic capacity is significantly lower after 48 h metformin treatment (HG: p < 0.0001; LG: p < 0.0001) (Fig. 3e). The absolute levels of normalized OCR and ECAR in each condition are included in supplementary information (Supplementary Fig. S2). The metabolic profiles based on basal levels of OCR and ECAR (Fig. 3f) show the differences and comparative shift of the cell lines in both glucose concentrations and metformin treatment. Untreated, SW948 are more glycolytic, while SW1116 are more aerobic. Following metformin treatment, both cell lines are less energetic, but perhaps more noteworthy is that SW1116 converges with SW948 at a more glycolytic phenotype.

### Glut1 is affected by growth media and metformin treatment

**Figure 4:**
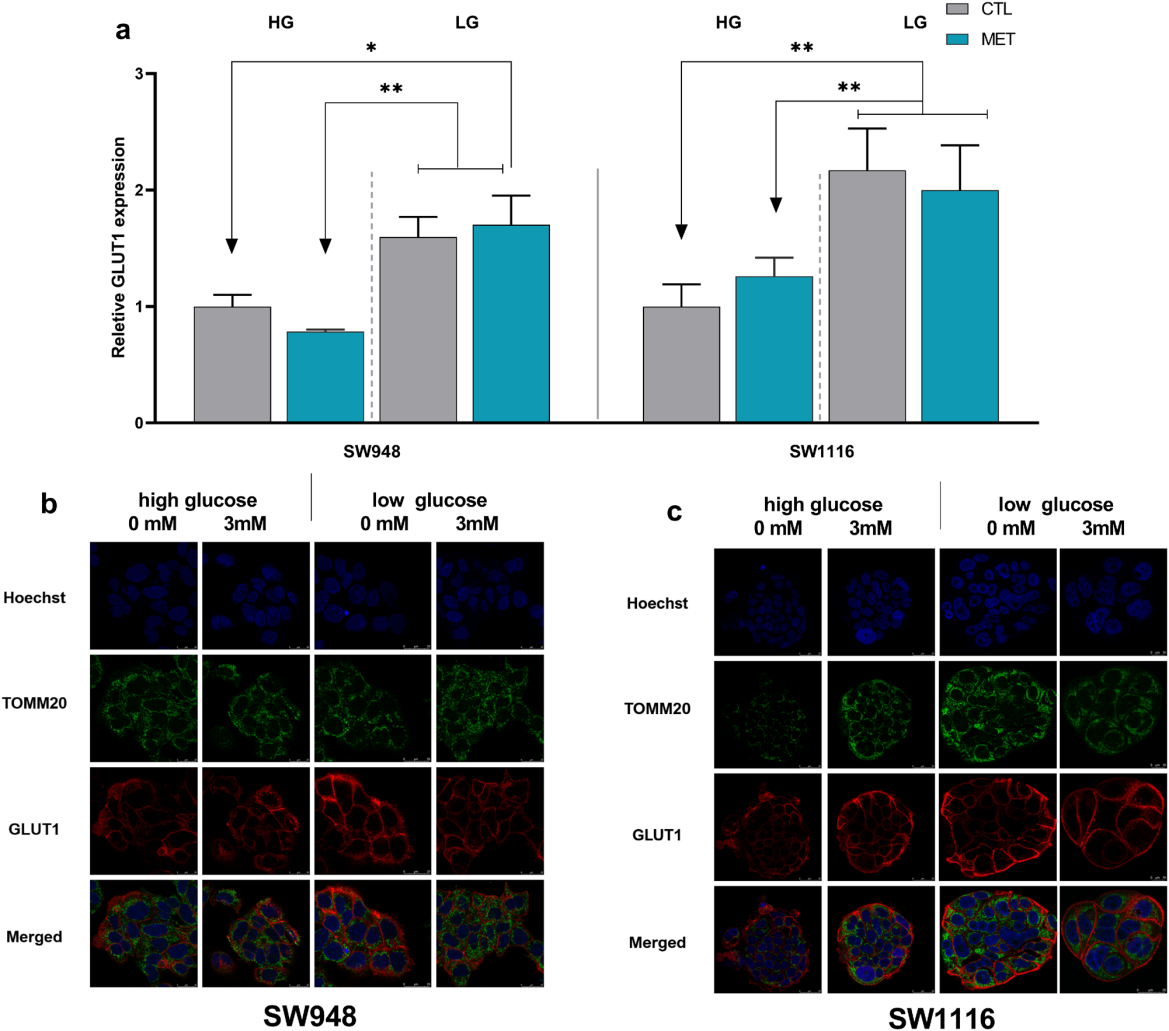
Protein expression analysis of GLUT1 in response to metformin treatment under different glucose culturing conditions. (**a**) Bars represent the relative GLUT1 fold inductions, measured by flow cytometry after 48 h metformin treatment in SW948 and SW1116, compared to respective controls at same glucose culturing conditions. Representative confocal images of (**b**) SW948 and (**c**) SW1116 using Hoechst for nuclei staining in blue, TOMM20 for the mitochondria in green, and GLUT-1 Antibody in red. Error bars denotes s.e.m. and statistical analysis was calculated using two-way ANOVA (*p < 0.05, **p < 0.01) (N = 3). *MET* Metformin, *CTL* control, *GLUT1* glucose transporter 1.

We analyzed protein expression of one of the major glucose import proteins, glucose transporter protein 1 (GLUT1), and how it correlates to the mitochondrial and glycolytic changes found in the metabolic flux analysis. GLUT1 significantly increases in SW948 cells under low glucose conditions compared to high glucose conditions. The corresponding change after metformin treatment is, however, not significant in these cells and follows the same pattern as glucose response (Fig. 4a). Similarly, in SW1116 grown in low glucose concentration, the GLUT1 expression increases compared to high glucose. Furthermore, no further increase is seen after metformin treatment (Fig. 4a). To verify location of GLUT1 expression and mitochondrial detection throughout the different growth conditions and metformin treatment we performed a multi-stained confocal analysis (Fig. 4b,c) for both cell lines, identifying GLUT1 in the cell plasma membrane during all conditions. We found no apparent change in mitochondrial morphology, nor in intracellular localization across the different treatments in either of the cell lines.

## Discussion

Here we show how growth conditions influence the metformin response in different cell lines, specifically concentration of glucose in the cell growth media. Using clinically achievable levels of metformin and changing the glucose concentration in the growth media for colorectal cell lines, SW948 and SW1116, resulted in changes in cell growth, metabolic flux, and protein expression. The glucose concentration directly influenced the growth rate in SW1116, where doubling time decreased in low glucose (5 mmol/L) media compared to high glucose (25 mmol/L), suggesting that glucose itself imposes an inhibitory growth effect in these cells. Comparatively, for SW948 there was no change in doubling rate between high glucose versus low glucose media. Most standard growth medias are formulated with high glucose content to avoid nutrient deprivation over time, however the finding that high glucose levels may mask true drug responses is not well considered.

We found that SW948 and SW1116 colon cancer cell lines were distinguishable in their ability to use their mitochondria, as shown in results from metabolic flux analysis. SW948 is, under non-stressed conditions (basal), running at maximum glycolytic capacity, further supported by our finding that under low glucose growth conditions these cells increased their expression of GLUT1 receptors. This could be a compensatory response for keeping up the high glucose flux through glycolysis with the lower amount of glucose available in the media as previously described^45^, thus supporting rapid proliferation. As SW948 resemble Warburg’s phenotype, i.e. rapid proliferation and are avid glucose consumers, they show an inability to maintain rapid proliferation under the mitochondrial-inhibiting effects of metformin even with an abundance of glucose, since glycolysis alone cannot support a high proliferation rate without mitochondrial contribution of biomolecules^2^. This is contrary to SW1116 cells that utilize their metabolic flexibility to overcome the effects of metformin by upregulating glycolysis, supporting their lower proliferation rate. In both glucose concentrations, SW1116 cells exhibit low to no additional respiration under CCCP stress; however, when treated with metformin, the relative rates of CCCP-uncoupled respiration to basal OCR increases significantly.

The abundance of glucose in culture media seems to affect the way both investigated metabolic phenotypes of cancer cells react to metformin. SW948 is more glucose dependent and able to thrive and grow exponentially in both glucose concentrations by altering their GLUT1 expression response to adapt to different conditions. SW1116 is more metabolically adaptable to glucose concentration considering its GLUT1 and metabolic flux response. Here we found that after treatment in high glucose concentration, SW1116 show a slight increase in GLUT1 which could be due to their attempt to shift their metabolism towards glycolysis upon mitochondrial stress induced by metformin. SW948 had reduced GLUT1 expression in high glucose with treatment. Both cell lines had increased GLUT1 expression in the low glucose concentration, however SW1116 had a much larger increase, in line with their significant increase in basal glycolysis. In general, SW948 keep their glycolytic dependency in low glucose compared to high glucose, but the cell proliferation ultimately slows, which could mean they have less adaptability to glucose concentration compared to SW1116. In high glucose and with metformin treatment, there is a significant drop in OCR due to the inhibition of the ETC in both cell lines; similar results have been shown elsewhere^46^. Inhibition of complex I causes a drop in the ATP-linked respiration under metformin treatment. This could mean there is an unmet need of ATP production, which could cause a drop in proliferation or an increase in glycolysis. A higher uncoupled respiration in the metformin treated samples points to remaining functionality in the mitochondria, but it is reserved for acute stress, as induced by the mitochondrial uncoupler CCCP. The SW1116 cells showed a decreased response to metformin when cultured in high glucose media. This could be due to a metabolic shift towards glycolysis in a likely attempt to compensate for the metformin-driven inhibition of the ETC and TCA and resulting decrease of ATP-production in the mitochondria. However, in both cell lines physiological glucose levels reveal an underlying concentration-dependent response to metformin. Three different assays were used here to assess the metformin response in our cell lines, presented by CCK-8 assay and direct cell counts (Fig. 2), and an Alamar blue assay (Supplementary Fig. S1). The Alamar blue assay, also known as resazurin assay, is commonly used to assess viability, however in our experimental set up did not correlate with counted cell numbers from the same assay wells. The CCK-8 assay which also measures metabolic activity, however with a different chemical reaction, showed good correlation to actual cell counts. We would thus advise caution when using viability assays that rely on metabolic activity to measure responses of metabolic treatments, without including a second confirming analysis as presented here by Hoechst cell counts (Fig. 2). This is well supported by previous studies who have found the same effect when using resazurin^47,48^. The sensitivity of cancer cells to drug treatments in different glucose concentrations is not only relevant for in vitro testing, but also clinically where many patients may present with T2D and elevated blood glucose levels, which has been associated with chemoresistance^49^.

Not all cancer cells exhibit Warburg metabolism. This is becoming increasingly documented, both in vitro and clinically^50–52^. The results we see here of the difference in response of cell lines with different metabolic phenotypes, could explain the lack of response in the many clinical trials studying metformin. If only metabolically compatible cancers will respond and there are no criteria for treatment based on this, then it is unlikely a clear response would be seen when studying a mixed-phenotype clinical cohort. We found that metformin treated SW1116 shift toward a more glycolytic profile resembling that of the SW948 cell line. Fast proliferation is what is being targeted by cytotoxic chemotherapy drugs and SW948 is documented as being more susceptible to these, exhibiting lower IC50 to both 5-fluorouracil and oxaliplatin (GDSC2)^53^. However, cytotoxic drugs do seem to be targeting more than just proliferation^54^, with one possible target being metabolic reprogramming. If this is the case, the metabolic shift of SW116 to be more like SW948 could then also result in increased vulnerability to chemotherapy. In this vein, metformin may be used a neoadjuvant agent in an effort to increase response^55–58^. If patient tumours are assessed for their metabolic phenotypes, either by metabolic analysis of biopsy tissue^59^ or advanced tumour imaging^60–62^, this can be directly translated to the clinic for improved course of treatment.

## Methods

### Cell culture, proliferation, and viability assessments

SW948 and SW1116 were purchased from ETCC and cultured under humidified conditions in a 5% CO_2_ incubator at 37 °C. The culture media DMEM contained no glucose, (Corning, New York, USA) and was supplemented with 10% fetal bovine serum (FBS) (BioWest, Nuaillé, France), 2 mM (0.584 g/L) l-glutamine (Corning, New York, USA), penicillin (100 U/mL) and streptomycin (100 µg/mL) (Merck Millipore Corporation, Burlington, USA). For glucose experiments the DMEM media was supplemented with 25 mmol/L or 5 mmol/L glucose (Sigma-Aldrich, St. Louis, USA) concentrations for high glucose (HG) and low glucose (LG) culture conditions, respectively. The cells were acclimated to the glucose levels by being cultured and passaged several times in the respective glucose concentrations prior to metformin experiments. *Proliferation:* Cells were seeded in 96-well plates in 25 mmol/L or 5 mmol/L glucose supplemented media at a density of 20,000 cells/well or 10,000 cells/well for SW1116 and SW948, respectively. The plates were placed in the Incucyte ZOOM system (Essen Bioscience, Newark, United Kingdom) and monitored for 9 days with phase contrast images captured every 2 h. Media was exchanged on days 2 and 7. Growth was measured by analyzing the confluence of the cells over time using the Incucyte ZOOM software and reported as percent of image area covered. Doubling times were calculated during their respective log phases: 24–74 h for SW948 and 50–100 h for SW1116. Statistical analysis was performed as described in methods using an unpaired Student’s t-test. *Viability:* Both cell lines were treated in 96-well plates at an initial seeding density of 10,000 cells/well using increasing concentrations of 1.5–24 mM of metformin hydrochloride (Sigma-Aldrich, St. Louis, USA) to determine the cellular viability after 48 h. Both Alamar blue (Resorufin) and CCK-8 (CCK-8; Dojindo Laboratories, Kumamoto, Japan) assays were carried out according to the manufacturer’s protocol to estimate cell viability using fluorescence (Ex: 540 nm, Em:590 nm) and absorbance (450 nm) respectively via SpectraMax Paradigm plate reader (Molecular Devices, San Jose, USA). Cells in the Alamar blue plates were post-stained with Hoechst 33,342 (5 µg/mL) after fixation using 4% paraformaldehyde for 30 min, upon imaging using Leica SP8 Florescence microscope (Leica Microsystems, Mannheim, Germany). Image analysis using density counting of the nucleus was performed using ilastik^63^. Statistical analysis was calculated using two-way ANOVA.

### Metabolic analysis

Mitochondrial respiration and glycolysis were measured using the Seahorse XF96e flux analyzer (Agilent Technologies, Santa Clara, USA). Cells were seeded in XF96e cell culture plates at a density of 20,000 cells/well or 10,000 cells/well for SW1116 and SW948, respectively. They were allowed to attach overnight before treatment with 3 mM metformin hydrochloride for 48 h. Prior to the mitochondrial respiration assay, culture media was exchanged for unbuffered, serum-free DMEM, composed of DMEM 8.3 g/L (D5030, Sigma-Aldrich, St. Louis, USA) pH 7.4, NaCl 1.85 g/L (Sigma-Aldrich, St. Louis, USA), 2 mM l-glutamine (Corning, New York, USA), and glucose (concentration dependent on condition as described in results) (Sigma-Aldrich, St. Louis, USA). For the glycolysis assays, the assay media contained no glucose. The plates were then incubated at 37 °C in a CO_2_-free incubator for 1 h prior to running the assay. Oxygen Consumption Rate (OCR) and ExtraCellular Acidification Rate (ECAR) were measured over 100 min (15 mix and measure cycles), with compounds being injected every 3 cycles. For the mitochondrial respiration assays, the following compounds (Sigma-Aldrich, St. Louis, USA) were injected sequentially (final concentrations in the wells): Oligomycin (3 μM), CCCP (0.25 μM), Rotenone (1 μM), and Antimycin A (1 μM). For the glycolysis assays, the following compounds (Sigma-Aldrich, St. Louis, USA) were injected sequentially (final concentrations in the wells): glucose (10 mM), oligomycin (3 μM), CCCP^64,65^ (0.25 μM), 2-deoxy-d-glucose (100 mM). Protein concentration was measured in each well for normalization using a Pierce BCA assay (ThermoFisher Scientific, Rockford, USA) according to manufacturer’s instructions. Statistical analysis was performed using one-way ANOVA.

### Flow cytometry quantification of Glut1

SW948 and SW1116 cells were treated with 3 mM of metformin for 48 h at a seeding density of 1.0 × 10^6^ cells per well using HG and LG media. After treatment, cells were trypsinized and washed twice with PBS before adding 4% Paraformaldehyde (PFA) fixation and incubating on ice. The fixed cells were subsequently incubated with GLUT1 primary antibody (Abcam, Cambridge, United Kingdom) at the concentration 1:500 for 1 h at room temperature. GLUT1 labelled cells were washed twice in PBS and labelled with Alexa fluor 647 conjugated Donkey anti-rabbit secondary antibody (Abcam, Cambridge, United Kingdom) for another 30 min before analysing with Accuri C6 flow cytometer (BD Biosciences, San Jose, USA).

### Confocal imaging

Both cell lines were seeded in Ibidi µ-Slide 8-well chambered coverslips (Ibidi GmbH, Munich, Germany) at a density of 30,000 cells/well and were allowed to attach overnight in HG and LG media. 3 mM metformin treatment was added for 48 h before the cells were washed with PBS and fixed using 4% PFA. After 1 h of incubation with 20% FCS blocking solution, the cells were incubated overnight with GLUT1 Alexa fluor 647 conjugated antibody (Abcam, Cambridge, United Kingdom) (1:1000 in blocking solution). The next day, the wells were washed again and the cells permeabilized using 0.5% triton X in PBS for 15 min at room temperature**,** before another incubation step using TOMM20 antibody (Abcam, Cambridge, United Kingdom) 1:1000 in blocking solution overnight. The next day, cells were counterstained with Hoechst 33342 (ThermoFisher Scientific, Rockford, USA) (15 µg/ml solution) for 2 min, then washed 3 times with PBS. The cells were then imaged on a Leica TCS SP8 confocal microscope (Leica Microsystems, Mannheim, Germany)**.**

### Statistical analysis

Statistical comparisons were made using GraphPad PRISM (version 8, GraphPad Software, Inc., USA) software with one-way or two-way ANOVA to determine significant differences between several treatment groups. Post-hoc corrections for multiple comparisons were applied according to recommendations by GraphPad for each experimental data set (viability: Dunnett; metabolic analysis: Sidak; flow cytometry: Tukey). A student’s unpaired t-test was employed when only two groups were compared. The number of biological replicates (N) are given in the figures and legends. Values that follow ± within the results section are standard deviation (s.d.).

## Supplementary Information

Supplementary Figure S1.

Supplementary Figure S2.

## Data Availability

The datasets generated during the current study are available on figshare, https://doi.org/10.6084/m9.figshare.13490271.
