# Author Correction: Metformin treatment response is dependent on glucose growth conditions and metabolic phenotype in colorectal cancer cells

**DOI:** 10.1038/s41598-022-06177-9

**Published:** 2022-01-28

**Authors:** Abdelnour H. Alhourani, Tia R. Tidwell, Ansooya A. Bokil, Gro V. Røsland, Karl Johan Tronstad, Kjetil Søreide, Hanne R. Hagland

**Affiliations:** 1 Department of Chemistry, Bioscience and Environmental Engineering, University of Stavanger, Stavanger, Norway; 2 Department of Biomedicine, University of Bergen, Bergen, Norway; 3 Department of Oncology and Medical Physics, Haukeland University Hospital, Bergen, Norway; 4 Department of Gastrointestinal Surgery, Stavanger University Hospital, Stavanger, Norway

Correction to: *Scientific Reports* 10.1038/s41598-021-89861-6, published online 18 May 2021

The original version of this Article contained an error in the Figure 3 (**f**) legend,

“(**f**) OCR vs ECAR from the mitochondrial stress test assay. Squares: SW948; Circles: SW116.”

now reads:

“(**f**) OCR vs ECAR from the mitochondrial stress test assay. Squares: SW1116; Circles: SW948.”

The original Article has been corrected.

