# Supplementary material for: Metformin treatment response is dependent on glucose growth conditions and metabolic phenotype in colorectal cancer cells
Source: Sci Rep. 2021 May 18;11:10487. doi: 10.1038/s41598-021-89861-6 (PMC8131751; doi:10.1038/s41598-021-89861-6)
Supplement: Supplementary file 1 — Supplementary Figure S1. [file 41598_2021_89861_MOESM1_ESM.pdf]

Metformin treatment response is dependent on glucose growth conditions and metabolic phenotype in colorectal cancer cells

Abdelnour Alhourani †[1], Tia Tidwell †[1], Ansooya A. Bokil [1], Gro V. Røsland PhD [2,3], Karl Johan Tronstad PhD [2], Kjetil Søreide MD, PhD [4], Hanne R. Hagland PhD [1*]

1. Department of Chemistry, Bioscience and Environmental Engineering, University of Stavanger, Stavanger, Norway
2. Department of Biomedicine, University of Bergen, Bergen, Norway
3. Department of Oncology and Medical Physics, Haukeland University Hospital, Bergen, Norway
4. Department of Gastrointestinal Surgery, Stavanger University Hospital, Stavanger, Norway

* Corresponding author: Associate professor H. R. Hagland PhD, Department of Chemistry, Bioscience and Environmental Engineering, University of Stavanger, Stavanger, Norway

E-mail: hanne.r.hagland@uis.no

Supplementary Figure S1.

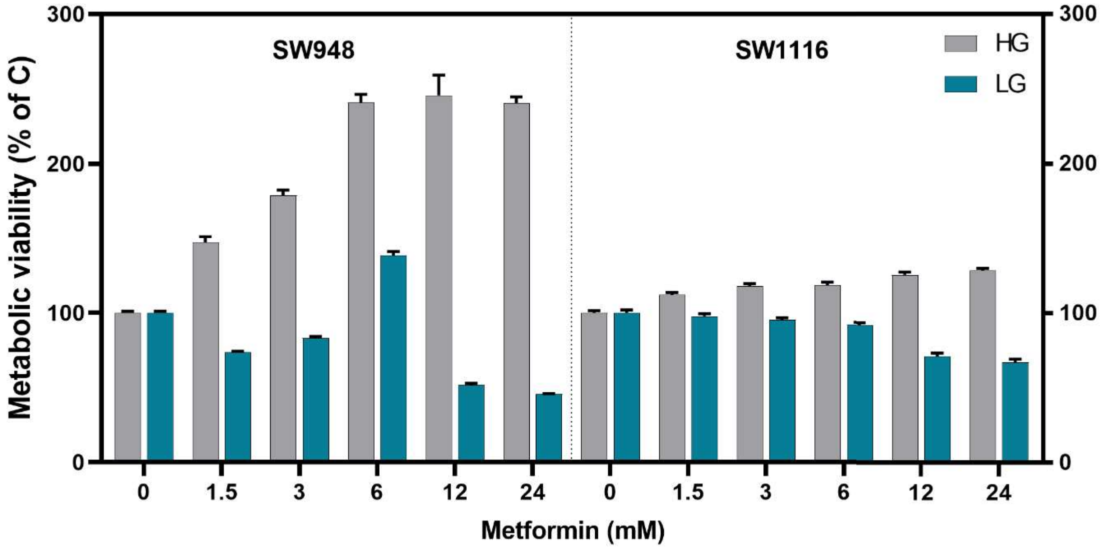

Figure S1. Metabolic viability assay by Alamar blue reagent after increasing metformin treatment. Alamar blue absorbance values of metformin treated samples are shown normalized to their own high (25 mmol/L) or low (5mmol/L) glucose control. Cells were seeded at 20 000 cells/well for SW1116 and 10 000/well for SW948, with increasing metformin concentrations added to each well and left for 48 hours. N=3 for all conditions. Error bars denotes s.e.m. Abbreviations: HG; high glucose, LG; Low glucose.
