# Supplementary material for: Metformin treatment response is dependent on glucose growth conditions and metabolic phenotype in colorectal cancer cells
Source: Sci Rep. 2021 May 18;11:10487. doi: 10.1038/s41598-021-89861-6 (PMC8131751; doi:10.1038/s41598-021-89861-6)
Supplement: Supplementary file 2 — Supplementary Figure S2. [file 41598_2021_89861_MOESM2_ESM.pdf]

# Metformin treatment response is dependent on glucose growth conditions and metabolic phenotype in colorectal cancer cells

Abdelnour Alhourani †[1], Tia Tidwell †[1], Ansooya A. Bokil [1], Gro V. Røsland PhD [2,3], Karl Johan Tronstad PhD [2], Kjetil Søreide MD, PhD [4], Hanne R. Hagland PhD [1*]

1. Department of Chemistry, Bioscience and Environmental Engineering, University of Stavanger, Stavanger, Norway
2. Department of Biomedicine, University of Bergen, Bergen, Norway
3. Department of Oncology and Medical Physics, Haukeland University Hospital, Bergen, Norway
4. Department of Gastrointestinal Surgery, Stavanger University Hospital, Stavanger, Norway

* Corresponding author: Associate professor H. R. Hagland PhD, Department of Chemistry, Bioscience and Environmental Engineering, University of Stavanger, Stavanger, Norway

E-mail: hanne.r.hagland@uis.no

Supplementary Figure S2.

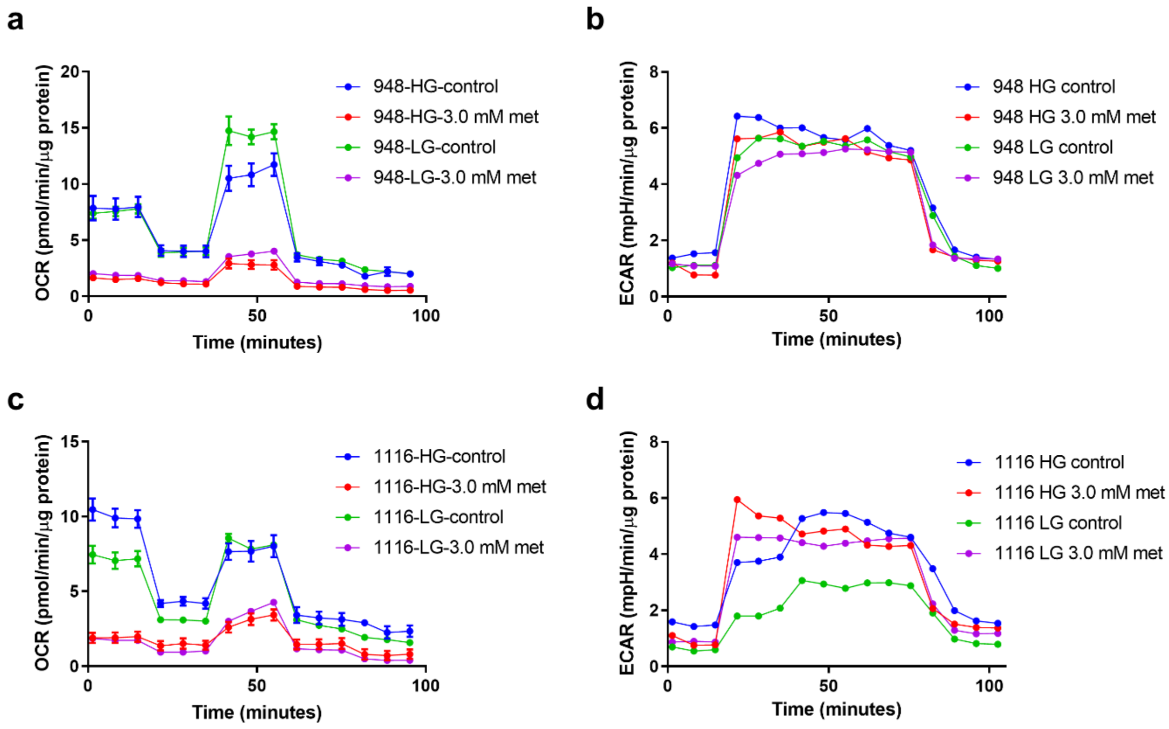


Figure S2. Oxygen consumption rates (OCR) and extracellular acidification rates (ECAR) traces from the mitochondrial and glycolysis stress test assays. All values are normalized to protein concentration from each individual well. Mean values ± s.e.m. are plotted. N=2-8 (a) SW948 OCR (b) SW948 ECAR

(c) SW1116 OCR (d) SW1116 ECAR.  Abbreviations: HG, high glucose (25 mmol/L); LG; Low glucose (5 mmol/L); met, metformin.
